# Novel deleterious mutation in *MYO7A*, *TH* and *EVC2* in two Pakistani brothers with familial deafness

**DOI:** 10.12669/pjms.35.1.98

**Published:** 2019

**Authors:** Bibi Sabiha, Johar Ali, Yasar Mehmood Yousafzai, Syed Adnan Haider

**Affiliations:** 1 Bibi Sabiha, Center for Genomic Sciences, Rehman Medical College, Phase-V, Hayatabad, Peshawar, KP, Pakistan; 2 Johar Ali, Center for Genomic Sciences, Rehman Medical College, Phase-V, Hayatabad, Peshawar, KP, Pakistan; 3 Yasar Mehmood Yousafzai, Institute of Basic Medical Sciences, Khyber Medical University, Peshawar, Pakistan; 4 Syed Adnan Haider, Center for Genomic Sciences, Rehman Medical College, Phase-V, Hayatabad, Peshawar, KP, Pakistan

**Keywords:** Deafness, *EVC2*, *MYO7A*, Next Generation Sequencing, TH

## Abstract

**Objective:**

In Pakistan, 74% of consanguineous marriages are among the first cousins. Continuity of consanguineous marriages over generations increases the risk of recessive diseases such as deafness. The objective of this study was to investigate genetic origin of Pakistani deaf brothers with parents of consanguineous marriage.

**Methods:**

DNA was extracted from the blood through Qiagen kit. Paired-end sequencing library was prepared according to protocol of Illumina’s TruSight Rapid Capture kit and TruSight Inherited Disease Panel. Library was normalized and used for Next Generation Sequencing through MiSeq. NGS data were analyzed using various bioinformatics tools.

**Results:**

Both brothers were found to have novel deleterious mutation in *MYO7A* (c.2476G>A) while the younger brother had additional novel deleterious mutation in *TH* (c.43C>T) and *EVC2* (c.2614C>T) genes.

**Conclusion:**

It is concluded that in addition to novel mutations in *MYO7A, TH* and *EVC2*, the *CDH23 and GJB2* can also be responsible for deafness in the family with consanguineous marriages.

## INTRODUCTION

The word consanguinity describe unions between couples who share at least one common ancestor.^1^ Consanguineous marriages are preferred in South Asia. According to the Pakistan Demographic and Health Survey (2012-2013), approximately 65% of marriages were consanguineous, out of which 74% were among first cousins.^2^ The recessive disorders show the highest association with consanguinity (78.8%).^3^ Consanguinity is a recognized high-risk factor among the etiological factors for deafness.^4^ Although environmental factors are involved in deafness, the genetic defects have been reported to play a major role contributing to estimated 60% of hearing loss (HL).^5^ The spectrum of genetic mutations involved in deafness vary amongst different populations.^6^ Most highly studied gene in different populations is GJB2 (OMIM: 121011) encoding gap junction protein, beta-2 and account for 50% of non-syndromic hearing loss (NSHL) throughout the world.^7^ Connexins are trans-membrane proteins that have a role in communication and transferring of ions and small signaling molecules between cells.^8^ This locus is already been reported in Pakistani and Mediterranean families supporting that *GJB2* is the main gene for inherited sensorineural deafness.^9^ More than 90 variants in *GJB2* have been reported and meta-analysis in different ethnic group confirmed a strong association of *GJB2* mutation with HL in different population.^10^

In addition to *GJB2*, >80 mutations in MYO7A (OMIM: 276903) encoding myosin VIIA have also been linked with syndromic and NSHL.^11^ *MYO7A* co-ordinates between the transduction channel and stereocilia membrane and interact with hair cells by associating with cadherin molecules. Lack of function within the cochlear hair cells leads to development of deafness.^12^ Similarly, *CDH23*, (OMIM: 605516*)* gene encoding Cadherin-23 showed mutation in both NSHL (DFNB12) and Usher syndrome type ID (USH1D).^13^ *CDH23* contain 69 exons and codes 3,354-amino-acid protein comprising 27 cadherin extracellular (EC) repeats, a transmembrane domain and a unique cytoplasmic domain. Function of *CDH23* has not yet been defined but it is hypothesized to be involved in cell-cell adhesion because of its similarity in structure with Epithelial Cadherin (E-Cadherin) which has role in homophilic cell-cell adhesion.^14^ Additionally, genetic variations in *TH* (OMIM: 191290) and *EVC2* (OMIM: 607261) encoding Tyrosine Hydroxylase and EvC Ciliary Complex Subunit 2 respectively, have also been reported in deafness.

It is hypothesized that the spectrum of genetic mutations in Pakistani population with hereditary hearing loss might be different from elsewhere. This study set out to use advanced Next Generation Sequencing (NGS), MiSeq (Illumina, San Diego, US), to determine the genetic variation of *GJB2*, *MYO7A*, *CDH23, TH* and *EVC2* in two Pakistani deaf brothers.

## METHODS

### Subject description and sample collection

The study was approved by the Institutional Review Board (IRB) at Rehman Medical Institute (RMI) Peshawar, Pakistan (Ethics approval no. DMR/RS/JA/CGS/01).

**Fig.1:**
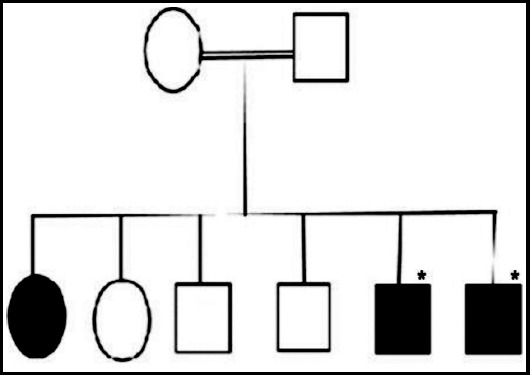
Pedigree of the affected family. Circles represent female and squares male family members. Affected individuals are denoted by shaded squares and circles. Asterisk shows the subjects underwent molecular study.

Two deaf brothers (15 and 23years old), were recruited for targeted gene sequencing after taking informed consent. Patient history revealed that their parents, two brothers and one sister were normal, however, one sister was deaf (Fig.1). Their parents were also second cousins and were not available for genetic study. The patients were clinically assessed in the ENT department of the RMI by a qualified ENT specialist. Examination of the ENT revealed no anatomical abnormality or signs of infection or inflammation. Pure Tone Audiometry (PTA) was performed which confirmed bilaterally sensorineural hearing defect with no difference between air and bone conduction. Subsequently the patients were labelled as ‘deaf’. Two ml peripheral whole blood sample was drawn from the subjects in EDTA tubes by phlebotomist using aseptic techniques.

### DNA extraction, quantification and normalization

DNA was extracted from blood samples through Qiagen DNA mini kit (Qiagen, Cat. no. 69504). It was then quantified with help of dsDNA high sensitivity kit (Qubit, Cat. no. Q32851) using Qubit fluorometer and normalized to 5ng/µL.

### Library preparation

According to Illumina’s TruSight Rapid Capture kit (Cat. no. FC-140-1103) and TruSight Inherited Disease Panel (Cat. no. FC-121-0205) protocol, libraries for paired-end sequencing were prepared from extracted DNA. TruSight Inherited Disease Panel covers 552 genes associated with severe, recessive pediatric onset disease.

### Data analysis

FASTQ files were separated by CASAVA and trimmomatic tool was used to filter the low quality reads (Q>30). The filtered reads were aligned to the reference genome (hg19/GRCh37) and Variant Calling Format (VCF) was produced through GATK’ Haplotype Caller tool. The VCF file was manually validated using Integrated Genomics Viewer (IGV) and analyzed on Illumina’s Variant Studio to check the deafness associated genes.

## RESULTS

The use of inherited disease genes sequencing panel identified the causative and novel variants in deafness related genes (GJB2, MYO7A, CDH23, TH and EVC2) in deaf brothers.

### Variants in GJB2 gene

Two variants C>T/T (rs7623, c.*1152G>A) and C>A/A (rs9237, c.*1067G>T) in GJB2 gene were found common in both brothers. In younger brother two additional variants in GJB2 gene were also identified which were A>G/G (rs7988691, c.*1277T>C), A>G/G (rs3751385, c.*84T>C) (Table-I).

**Table-I:** Variation detected in GJB2, MYO7A and CDH23 genes in both brothers.

Genes	Chr:Pos	Ref/Alt	Identifier	HGVSc	Consequences	Elder brother	Coverage Depth	Younger brother	Allele Frequency (ExAC)	Coverage Depth
GJB2	13:20761888	C>T/T	rs7623	c.*1152G>A	3_prime_UTR_variant	Homo^1^	T: 46 (100%, 30+,16-)	Homo^1^	N/A	G: 1 (1%, 1+, 0-) T:106 (99%, 62+, 44-)
GJB2	13:20761973	C>A/A	rs9237	c.*1067G>T	3_prime_UTR_variant	Homo^1^	A: 44 (100%, 25+, 19-)	Homo^1^	N/A	A: 74 (100%, 40+, 34-)
GJB2	13:20761763	A>G/G	rs7988691	c.*1277T>C	3_prime_UTR_variant	No change	-	Homo^1^	N/A	G: 22 (100%, 9+, 13-)
GJB2	13:20762956	A>G/G	rs3751385	c.*84T>C	3_prime_UTR_variant	No change	-	Homo^1^	N/A	G:30 (100%, 15+, 15-)
MYO7A	11:76853783	T>C/C	rs1052030	c.47T>C	missense_variant	Homo^1^	C: 4 (100%, 2+, 2-)	Homo^1^	0.43	C: 10 (100%, 6+, 4-)
MYO7A	11:76919478	C>A/A	rs948962	c.5860C>A	missense_variant	Homo^1^	A: 25 (100%, 12+, 013-)	Homo^1^	0.47	A: 77 (100%, 39+, 38-)
MYO7A	11:76912636	A>T/T	rs2276288	c.4996A>T	missense_variant	Homo^1^	T: 10 (100%, 4+, 6-)	Homo^1^	0.54	T: 37 (100%, 16+, 21-)
CDH23	10:73501556	G>A/A	rs1227051	c.4723G>A	missense_variant	Homo^1^	A: 22 (100%, 11+, 11-)	Homo^1^	0.77	A:48 (96%, 25+, 23-)
										C:2 (4%, 0+, 2-)
CDH23	10:73270906	T>T/C	rs3802720	c.366T>C	synonymous_variant	Het^2^	C: 9 (36%, 4+, 5-)	No change	0.67	-
							T: 16 (64%, 8+, 8-)			
CDH23	10:73377314	C>C/G, G/G	rs7903772	c.1134+164C>G	intron_variant	Het^2^	C: 11 (50%,5+, 6-)	Homo^1^	N/A	G:43 (100%, 19+, 24-)
							G: 11 (50%,5+, 6-)			
CDH23	10:73377330	T>T/C	rs6480536	c.1134+180T>C	intron_variant	Het^2^	C: 11 (55%, 5+, 6-)	No change	N/A	-
							T: 9 (45%, 4+, 5-)			
CDH23	10:73434888	G>G/C, C/C	rs1227049	c.1469G>C	missense_variant	Het^2^	C:21 (57%, 9+, 12-)	Homo^1^	0.19	C:105 (100%, 51+, 54-)
							G: 16 (43%, 8+, 8-)			
CDH23	10:73455201	T>T/C	rs3752752	c.2316T>C	synonymous_variant	Het^2^	C: 12 (44%, 6+, 6-)	No change	0.63	-
							T: 15 (56%, 7+, 8-)			
CDH23	10:73537978	C>C/T, T/T	rs10762480	c.5100C>T	synonymous_variant	Het^2^	C: 14 (70%, 8+, 6-)	Homo^1^	0.18	T:47 (100%, 24+, 23-)
							T: 6 (30%, 3+, 3-)			
CDH23	10:73544086	G>G/A, A/A	rs3802711	c.5411G>A	missense_variant	Het^2^	A: 13 (52%, 7+, 6-)	Homo^1^	0.15	A:50 (100%, 27+, 23-)
							G: 12 (48%, 5+, 7-)			
CDH23	10:73550969	G>A/A	rs10466026	c.6130G>A	missense_variant	No change	-	Homo^1^	0.31	A:85 (100%, 41+,44-)

1 Homozygous,

2 Heterozygous

### Variants in MYO7A gene

Three common missense variation T>C/C (rs1052030, c.47T>C), C>A/A (rs948962, c.5860C>A), A>T/T (rs2276288, c.4996A>T) in MYO7A were found in both brothers (Table-I).

### Variants in CDH23 gene

The missense variant which is common in both brothers in CDH23 gene is G>A/A (rs1227051, c.4723G>A). C>C/G, G/G (rs7903772, c.1134+164C>G), G>G/C, C/C (rs1227049, c.1469G>C), C>C/T, T/T (rs10762480, c.5100C>T), G>G/A, A/A (rs3802711, c.5411G>A) in CDH23 were also identified in both brothers. Furthermore, G>A/A (rs10466026, c.6130G>A) in CDH23 were only found in younger brother. Three additional variants T>T/C (rs3802720, c.366T>C), T>T/C (rs6480536, c.1134+180T>C), T>T/C (rs3752752, c.2316T>C) were found only in elder brother (Table-I).

### Novel variants

In the same way novel variants in TH (G>G/A, c.43C>T), EVC2 (G>G/A, c.2614C>T) were found in younger brother. One novel missense variation MYO7A (G>A/A, c.2476G>A) was also found in MYO7A in both brothers (Table-II).

**Table-II:** Novel variation detected in EVC2, TH, and MYO7A genes in both brothers.

Gene	Variant	HGVSc	Chr	Consequence	SIFT	PolyPhen	Elder brother	Younger brother
EVC2	G>G/A	c.2614C>T	4	missense variant	Deleterious (0.04)	Probably damaging (0.997)	No change	Het^2^
TH	G>G/A	c.43C>T	11	missense variant	Deleterious (0)	Probably damaging (0.999)	No change	Het^2^
MYO7A	G>A/A	c.2476G>A	11	missense variant	Deleterious (0)	benign (0.258)	Homo^1^	Homo^1^

1 : Homozygous

2 : Heterozygous

## DISCUSSION

Most frequently implicated genes in autosomal recessive non-syndromic hearing loss (ARNSHL) are *GJB2* followed by *SLC26A4 (*OMIM: 605646*)*, *MYO15A* (OMIM: 602666), *OTOF* (OMIM: 603681), and *CDH23*.^15^ Impact of *GJB2* on HL has been determined previously in European (35delG, 167delT).^16^

This study evaluated the association of *GJB2*, *MYO7A*, *CDH23*, *TH* and *EVC2* with deaf in Pakistani brothers. The variations were in 3’-UTR of *GJB2*, coding region of *MYO7A* and *CDH23* (Table-I). Common homozygous variants in both brothers in 3’-UTR of *GJB2* were (rs9237, c.*1067G>T) and (rs7623, c.*1152G>A) which had already been reported in Portuguese family. These findings support the same in Portuguese family.^17^ Hence this study strongly suggests that these two homozygous variant in *GJB2* are responsible for deafness in these patients.3’-UTR often contain regulatory elements, variation in this region affect the spatial and temporal gene expression. So the variation in 3’-UTR of *GJB2* may also affect expression of *GJB2* resulting to deafness.

The genetic variation in deafness varies amongst different populations. Deletion of T at position 167 (mutation 167delT) and G at position 35 of the *GJB2* (mutation 35delG) results in premature chain termination in Non-syndromic Neurosensory Autosomal Recessive Deafness (NSRD) in Italian patients.^9^ These findings represents that the variation in *GJB2* affect the normal function of *GJB2* leading to HL or deafness. Syndromic and non-syndromic hearing impairment are caused by mutation within *MYO7A* in humans. More than 80 *MYO7A* mutations have been identified and are known to inherit in a recessive manner.^11^ This gene encode protein, the myosin VIIA, expressed in inner ear, retina, testis and lungs. Lack of adequate myosin VIIA function within the cochlear hair cells leads to development of deafness.^18^ In present study we report homozygous novel mutation in coding region of *MYO7A* (G>A/A, c.2476G>A) in both brothers. Apart from this novel mutation, this study also reports three other missense variants in *MYO7A* in both brothers. Three of these variants are reported in gentamicin-induced vestibular dysfunction (rs948962),^19^ malignant melanoma (rs2276288 and rs1052030)^20^ and are not reported in deafness. To our knowledge, this is the first study to report these missense variants in deafness.

Common variants, G>A/A, (rs1227051, c.4723G>A) in *CDH23* were identified in both brothers in homozygous form which had also been reported in Korean HL patients as a non-synonymous mutation.^21^ The same variation is presumed as polymorphism in Japanese population with NSHL.^22^ Therefore, the present study is an agreement with previous studies. Three additional variants T>T/C (rs3802720, c.366T>C), T>T/C (rs6480536, c.1134+180T>C), T>T/C (rs3752752, c.2316T>C) in *CDH23* were found only in elder brother. It is suggested that the three additional variations in *CDH23* might be in favor of more deafness in elder brother because his hearing ability was less than the younger brother.

In addition, Dopamine (DA) modulate amino acid neurotransmitter including GABA and has a role in auditory pathways and low-level secretion of GABA leads to deafness.^23^ *TH* is the rate limiting enzyme in dopamine synthesis and in return it is regulated by feedback mechanism.^24^ Variation found in *TH* (c.43C>T) possibly affect the hearing ability. Apart from that, this study also identified novel deleterious mutation in *EVC2* gene (c.2614C>T) only in younger brother which has already been known to be involved in Pakistani family with EVC syndrome and profound deafness.^25^ Therefore, it is concluded that these novel mutation may be of functional consequences on the causative variants and help in increasing the severity of the deafness. We have shown for the first time in Pakistan the novel mutations in deaf patients.

### Limitations of the study

Firstly, it reports only two brothers of the family. We could not test the apparently unaffected members of the family and therefore cannot comment upon the zygosity of the genetic mutations. Secondly, the sequence analysis was not validated by Sanger Sequencing. Although there is no consensus amongst the scientific community on the need to validate NGS results with Sanger sequencing.^26^ Lastly, no mechanistic studies were performed to assess the precise role of these novel variants in the pathogenesis of deafness.

## CONCLUSION

This study identified variations in *GJB2*, *MYO7A*, *CDH23*, *TH* and *EVC2* in deaf brothers. The three additional variations in *CDH23* might be in favor of profound deafness in elder brothers as he was deafer comparatively. Therefore, it is concluded that all the identified variants in this study may contribute to disease pathology. This pilot study is a proof of principle for high level study for deafness profiling in Pakistani population, which will be helpful in genetic counseling and to arrange marriages out of family to prevent genetic disease. It is also concluded that the family with recessive diseases should conduct the premarital genetic testing before the consanguineous marriages. The identified variants need to be confirmed by Sanger sequencing or NGS on large sample size.

### Author`s Contribution

**BS** conducted the experiment and manuscript writing.

**JA** secured funding, designed the study and edited the manuscript.

**SAH** did data analysis of the study.

**YMY** did review and final approval of manuscript.
